# Radiosensitisation of Head and Neck Cancer Cells to Protons of Increasing LET Through Targeting DNA Double Strand Break Repair

**DOI:** 10.3390/cells15100879

**Published:** 2026-05-12

**Authors:** Elizabeth R. Dufficy, Amalia Goula, Emma Melia, Abigail Bellamy, Jason L. Parsons

**Affiliations:** 1 Department of Cancer and Genomic Sciences, University of Birmingham, Edgbaston, Birmingham B15 2TT, UK; 2 School of Physics and Astronomy, University of Birmingham, Edgbaston, Birmingham B15 2TT, UK

**Keywords:** DNA damage repair, head and neck cancer, proton beam therapy, radiobiology, radiotherapy

## Abstract

**Highlights:**

**What are the main findings?**

- Inhibition of DNA double strand break repair, particularly through ATM and DNA-Pkcs, can significantly sensitise head and neck squamous cell carcinoma models to proton beam therapy.
- Radiosensitisation of head and neck squamous cell carcinoma models to ATM or DNA-Pkcs inhibition is independent of proton linear energy transfer.

**What are the implications of the main findings?**

- Targeting DNA double strand break repair is an effective strategy to enhance the efficacy of proton beam therapy.
- ATM and DNA-Pkcs inhibitors in combination with proton beam therapy has the potential to improve treatment of head and neck cancer patients.

**Abstract:**

The use of proton beam therapy (PBT), as a more precision-targeted radiotherapy technique, is increasing in the treatment of head and neck squamous cell carcinoma (HNSCC). PBT benefits from the precise delivery of the radiation dose to the tumour via the Bragg peak. However, challenges still remain in the treatment of HNSCC with radiotherapy, particularly with tumour radioresistance and recurrence, requiring strategies leading to radiosensitisation. There are added complexities with the use of PBT given the increase in linear energy transfer (LET) at and around the Bragg peak, which can cause an altered cellular response compared to low-LET radiation. Nevertheless, targeting the cellular DNA damage response is considered an important strategy to enhance tumour cell killing caused by radiotherapy. Therefore, using specific inhibitors against the protein kinases ataxia telangiectasia mutated (ATM), ataxia telangiectasia and Rad3-related (ATR) and the DNA-dependent protein kinase catalytic subunit (DNA-Pkcs), we investigated their impact in radiosensitising HPV-negative HNSCC cells to PBT of increasing LET. We demonstrate that inhibitors against ATR (AZD6738), and particularly ATM (AZD1390) and DNA-Pkcs (AZD7648), could significantly decrease clonogenic survival of HNSCC cell lines following PBT at both low and relatively high LET (~2 keV/µm and ~8 keV/µm, respectively). We confirmed that the inhibitors in combination with PBT led to DSB persistence through neutral comet assays and monitoring γH2AX/53BP1 foci. We also show that this strategy can enhance the sensitivity of patient-derived organoids of HNSCC to PBT of both low and high LET, highlighting this as a strategy which should be exploited further.

## 1. Introduction

Head and neck squamous cell carcinoma (HNSCC) remains a global public health concern due to the high incidences of ~890,000 new cases per year, plus a death rate of ~450,000 cases per year [1]. The throat, larynx, nose, sinuses, and mouth are the sites where HNSCCs develop, mainly attributed to excessive tobacco and alcohol use, together with high-risk (type-16/18) human papillomavirus (HPV) infection. Radiotherapy, predominantly using X-rays, continues to be a key modality for the treatment and management of HNSCC, although adverse side effects, tumour radioresistance and recurrence are a common problem. Furthermore, platinum-based agents such as cisplatin or carboplatin are often administered in combination with radiotherapy in the treatment of HNSCC, although again, intrinsic or acquired resistance is observed [2,3]. HPV-positive tumours are well-known to display an improved response to radiotherapy and chemotherapy, compared to HPV-negative disease, largely due to DNA repair deficiencies [4,5,6]. Hence, a major focus for the field is on strategies to overcome the radioresistance in HPV-negative HNSCC. Proton beam therapy (PBT) is increasingly being utilised for treatment of HNSCC particularly, as the dose of radiation can be more accurately delivered to the tumour site via the existence of the Bragg peak, which spares the associated normal tissues and organs at risk [7]. However, PBT creates biological and clinical uncertainties due to increases in linear energy transfer (LET) that exist at and around the Bragg peak where the bulk of the dose of radiation is deposited.

The therapeutic effect of radiotherapy in terms of tumour cell killing is largely driven through the induction and persistence of DNA damage, particularly DNA double strand breaks (DSBs) and complex DNA damage (CDD), which is defined as two or more DNA lesions within one to two helical turns of the DNA. Whilst the majority of DNA damage generated by low-LET radiation is isolated and therefore relatively simple for cells to repair, increases in LET lead to elevated levels in DNA damage complexity that can drive an enhanced therapeutic response. Nevertheless, the induction of DNA damage leads to the activation of the cellular DNA damage response (DDR), where specific proteins and pathways exist to recognise and repair the damage [8,9]. Of particular note is the DNA DSB repair pathways, namely non-homologous end-joining (NHEJ) that is activated throughout the cell cycle, and homologous recombination (HR) which is employed during S/G2 cell cycle phases when a sister chromatid is available as a repair template. The key to the signalling and repair of DSBs lies in a cascade of protein post-translational modifications, particularly phosphorylation initiated by the protein kinases ataxia telangiectasia mutated (ATM), ataxia telangiectasia and Rad-3-related (ATR) and DNA-dependent protein kinase catalytic subunit (DNA-Pkcs). Indeed, a key step is the phosphorylation of the histone H2AX variant (forming γH2AX), which is used as a surrogate marker of DSBs. Despite this, the roles of ATM, ATR and DNA-Pkcs in the repair of DSBs of increased complexity generated by higher LET radiation is currently unclear.

Radiosensitisation strategies are increasingly being sought to enhance the efficacy of the radiation whilst displaying a good safety profile. As previously mentioned, platinum-based agents have been used as radiosensitisers in HNSCC for over five decades, although other treatments such as anti-metabolites, inhibitors against EGFR, and cyclin-dependent kinases plus immunotherapies continue to be investigated [10,11]. In general, targeting the cellular DDR is considered a viable strategy that can enhance the efficacy of radiotherapy in cancer cell killing given that DNA is a key therapeutic target. In HNSCC, there is accumulating evidence specifically with X-ray radiation that this strategy can decrease the survival of HNSCC cells in vitro and suppress the growth of HNSCC tumour in vivo [12,13,14]. For example, DNA-Pkcs inhibitors KU-0060648, KU-57788 and IC87361 have shown increased X-ray radiosensitivity in several HNSCC cell lines [15,16]. Similarly, inhibitors of ATM (GSK635416A) and ATR (AZD6738 and VE-821) [15,17,18,19] have shown increased cell killing of HNSCC cells to X-rays. However, the potential for these DSB repair inhibitors to sensitise HNSCCs in response to PBT, particularly of increasing LET, is less clear. We have previously shown that both clonogenic survival and growth of 3D spheroids of HNSCC cells could be significantly reduced following treatment with inhibitors of ATR (AZD6738), but more so with inhibitors of ATM (AZD1390), and DNA-Pkcs (AZD7648), to low-LET PBT as well as X-rays [19]. Interestingly, there is controversial evidence to suggest that PBT may drive a higher dependence on the utilisation of HR coordinated by ATR (only demonstrated in lung cancer cells), which may be a consequence of the increase in LET that leads to CDD formation (reviewed in [7]). However, other evidence, albeit using significantly higher LET carbon ion therapy, has indicated that similar to low-LET X-rays, NHEJ dependent on DNA-Pkcs is still the major DSB repair pathway utilised. With this conflicting evidence, it is important to understand further which enzymes, and therefore the subsequent inhibitors against these, that are able to effectively sensitise to PBT across the Bragg peak where there is an increase in LET in order for any potential clinical strategies for patient benefit to be optimised.

In this study, we have directly analysed the effect of potent and specific inhibitors of ATM, ATR and DNA-Pkcs on the sensitivity of HPV-negative HNSCC cells and patient-derived organoids to PBT at both the entrance dose and closer to the Bragg peak, corresponding to low and relatively high LET, respectively. Our results generally demonstrate that there is no dramatic change in the radiosensitisation profiles through the inhibition of these protein kinases with increasing PBT LET, but where inhibitors of ATM and DNA-PKcs are the most potent radiosensitisers and which lead to persistent DNA DSB formation that drives tumour cell killing.

## 2. Materials and Methods

### 2.1. Cell and Patient-Derived Organoid Culture

Laryngeal squamous cell carcinoma cells (UMSCC12 and UMSCC17A) were kindly provided by Prof T. Carey, University of Michigan, USA. Cells from hypopharyngeal squamous cell carcinoma (FaDu) and submaxillary gland carcinoma (A253) were purchased from ATCC (Teddington, UK). Authentication of all cell lines was performed in our laboratory by STR profiling. Cells were regularly cultured as monolayers in 5% CO_2_ at 37 °C as previously described [19], using Dulbecco’s Modified Eagle Medium (DMEM) supplemented with 10% foetal bovine serum, 2 mM L-glutamine, 1× penicillin-streptomycin and 1× non-essential amino acids, except for FaDu cells and Detroit 562 cells which were cultured in Modified Eagle Medium (MEM).

Patient-derived organoids of HPV-negative HNSCC (HN041, HN080 and HNP155) with known molecular status were generated and cultured as previously described [20,21]. Key characteristics are described in Table S1.

### 2.2. Proton Irradiations

Cells were irradiated with PBT using a horizontal, passive-scattered proton beam line of 28 MeV maximal energy at the University of Birmingham Physics Department at a dose rate of ~5 Gy/min. Entrance dose, low-LET protons corresponding to an LET of 2.6 keV/µm were used, along with a PMMA degrader to position cells closer to the Bragg peak at an LET of 7.8 keV/µm, as previously described [22].

### 2.3. Clonogenic Assays

A defined number of cells were seeded into 35 mm dishes in triplicate and allowed to attach for ~16 h. Cells were then treated with inhibitors against ATM (AZD1390; ATMi, 10 nM), ATR (AZD6738; ATRi, 1 µM) or DNA-Pkcs (AZD7648; DNA-Pkcsi, 1 µM) for 1 h prior to irradiation. Inhibitors were kindly provided by AstraZeneca UK, and concentrations effective in kinase inhibition were determined as previously described [19]. Following treatment with PBT, the media was removed and fresh media added. The plating efficiencies for all the HNSCC cells were ~15–20%. To accommodate for cell death, increasing cell numbers were plated for increasing doses of PBT. After 7–14 days, colonies were fixed and stained with 6% glutaraldehyde and 0.5% crystal violet for 30 min. Colonies were washed to remove excess stain, left to air dry overnight, and the GelCount colony analyser (Oxford Optronics, Oxford, UK) was used to determine the numbers of colonies where settings were optimised for individual cell lines based on inclusion of distinct colonies of specific size and intensity, although the same settings were used across the various treatments. The surviving fraction was determined using the numbers of colonies per treatment level versus colonies that appeared in the untreated control. Results were accumulated from at least three independent biological experiments.

### 2.4. Organoid Viability Assays

In order to determine organoid sensitivity to X-rays and PBT, 1000 cells were seeded in a 5 µL dome of Cultrex RGF basement membrane extract (Bio-Techne, Minneapolis, MN, USA) in clear-bottom, black-walled 96-well plates and incubated for 5 days to allow organoids to form. Organoids were then treated with inhibitors against ATM (AZD1390; ATMi, 200 nM) or DNA-Pkcs (AZD7648; DNA-Pkcsi, 2 µM) for 4 h. DMSO and hydrogen peroxide (10 mM) were used as a vehicle-only control and positive control, respectively. Organoids were irradiated with X-rays (CellRad X-ray irradiator, Faxitron Bioptics, Tucson, AZ, USA) or PBT (both 2 or 4 Gy), and after 4 days, viability was determined using CellTiter Glo (Promega, Madison, WI, USA). The viability of organoids was calculated in treated samples compared to the DMSO control (the positive control was subtracted from both samples).

### 2.5. Enzyme-Modified Neutral Comet Assay

The enzyme-modified neutral comet assay was employed to detect the level of DSBs and CDD, as we recently described [22]. To simplify, after cells embedded in agarose were lysed, these were washed three times with enzyme reaction buffer (40 mM HEPES-KOH, 100 mM KCl, 0.5 mM EDTA and 0.2 mg/mL BSA, pH 8.0) and then incubated with either buffer alone (mock treated; revealing levels of DNA DSBs) or with buffer containing 5 pmol OGG1, 6 pmol NTH1 and 0.6 pmol APE1 (enzyme treated; revealing levels of DNA DSBs plus CDD) for at least 1 h at 37 °C in a humidified chamber. The DNA was allowed to unwind by placing slides in cold electrophoresis buffer (1× TBE buffer (pH 8.3)) in the dark for 25 min, followed by DNA migration through electrophoresis at 25 V, ~20 mA for 25 min. The slides were subsequently washed three times with 1 × PBS, dried overnight, then rehydrated for 30 min in water (pH 8.0). DNA staining was performed with SYBR Gold (Life Technologies, Paisley, UK) diluted 1:10,000 in water (pH 8.0) for 30 min, after which the slides were again air-dried overnight. For analysis, 50 cells per slide were scored in duplicate using the Komet 6.0 image analysis software (Andor Technology, Belfast, Northern Ireland). DSBs and CDD were expressed as % tail DNA values averaged from at least three independent, biological experiments.

### 2.6. Immunofluorescence Microscopy

Quantification of γH2AX and 53BP1 foci accumulating at DSBs was performed as previously described [20]. In brief, HNSCC cells were seeded on 13 mm coverslips and grown until ~70–80% confluent, irradiated with a fixed dose of PBT at 4 Gy, the media was replaced and cells incubated for up to 24 h in 5% CO_2_ at 37 °C to stimulate DNA repair. Cells on coverslips were then washed with PBS at room temperature for 5 min and fixed using 10% formalin for 10 min. Cell permeabilization was performed using 0.2% Triton X-100 in PBS for 10 min; this was followed by washing three times with 0.1% Tween-20 in PBS for 10 min, then blocking with 2% BSA in PBS for 30 min at room temperature to avoid non-specific staining. All incubations were performed on a rocking platform. Antibodies raised against γH2AX (05-636; Merck-Millipore, Watford, UK) or 53BP1 (A300-272A; Bethyl Labs, Montgomery, TX, USA) were diluted in 2% BSA and coverslips were incubated overnight at 4 °C. Coverslips were washed three times with PBS and then incubated with either goat anti-mouse Alexa Fluor 555 (A21422) or goat anti-rabbit Alexa Fluor 488 secondary antibodies (A11008; Thermo Fisher Scientific, Waltham, MA, USA) in 2% BSA for 1 h at room temperature in the dark. After washing with PBS for 10 min on a rocking platform, coverslips were mounted on a microscope slide using Fluoroshield containing DAPI (Sigma-Aldrich, Gillingham, UK). Cells were examined using an Olympus BX53 fluorescent microscope (Evident Europre GmbH, Stansted, UK) with a CAM-ORCA-FLASH4.0LTPlus Digital Hamamatsu sCMOS camera. CellSens Dimension Expandable Imaging software (version 4.3) was used to capture images (~20 images/cell line/antibody).

### 2.7. Statistical Analysis

All experiments were performed in at least triplicate as separate independent, biological experiments. The CFAssay for R package was used for statistical analysis of clonogenic survival data [23], where the linear-quadratic model (LQ model) is used to compare different treatment responses across increasing radiation doses. In order to calculate dose enhancement ratios at a surviving fraction of 0.37 (DER_37_) from linear-quadratic fitting, the doses required to achieve the same surviving fraction in the DMSO versus the inhibitor treated cells were calculated. Statistical analysis of the levels of DSB or CDD through enzyme-modified neutral comet assays or immunofluorescence microscopy was performed using either a one-sample or two-sample *t*-test.

## 3. Results

### 3.1. Inhibition of ATM and DNA-Pkcs Enhances Sensitivity of HNSCC Cells to Low- and High-LET PBT

**Figure 1:**
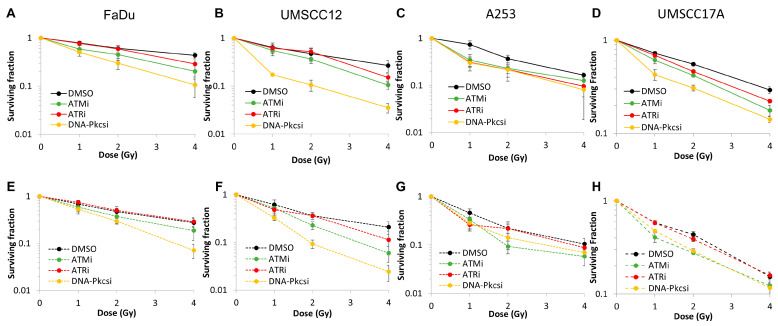
Inhibition of ATM and DNA-Pkcs drives increased sensitivity of HNSCC cells to both low- and relatively high-LET PBT. (**A**) FaDu, (**B**) UMSCC12, (**C**) A253 and (**D**) UMSCC17A cells were pretreated with inhibitors against ATM (10 nM), ATR (1 µM) and DNA-Pkcs (1 µM) or DMSO as a vehicle-only control, for 1 h prior to irradiation. Cells were exposed to increasing doses of (**A**–**D**) low-LET PBT or (**E**–**H**) relatively high-LET PBT, and clonogenic survival of cells was determined from three biologically independent experiments. Shown is the mean surviving fraction ± S.E.

We previously optimised concentrations of inhibitors against ATM (AZD1390), ATR (AZD6738) and DNA-Pkcs (AZD7648) that are effective in suppressing their target protein in HNSCC cell lines following PBT, through monitoring phosphorylation by immunoblotting indicative of kinase activation [19]. Using these inhibitor concentrations, we comparatively analysed the radiosensitivity of four HNSCC cell lines (FaDu, UMSCC12, A253 and UMSCC17A) irradiated with PBT at either the entrance dose at low LET (2.6 keV/µm), or with cells positioned closer to the Bragg peak at a relatively higher LET (7.8 keV/µm). We show that clonogenic survival of HNSCC cells was significantly reduced in the presence of inhibitors against either ATM, ATR or DNA-Pkcs versus the DMSO control following low-LET PBT (Figure 1A–D, Table 1 and Figures S1–S5). Dose enhancement ratios calculated at 37% survival (DER_37_; Table 2) revealed that inhibition of DNA-Pkcs is the most effective (1.76–2.69), compared to ATM (1.15–1.58) or ATR (0.79–1.77). Using relatively high-LET PBT delivered at the Bragg peak, we observed a similar profile of radiosensitisation of the HNSCC cells using the three inhibitors. Indeed, the greatest radiosensitisation was observed with inhibition of DNA-Pkcs (DER_37_ = 1.50–2.25), followed by ATM (1.41–1.66). Minimal radiosensitisation was observed in response to ATR inhibition (0.92–1.12). These results therefore suggest that DNA-Pkcs inhibition is the most effective strategy in reducing the survival of HNSCC cells in response to PBT, which is not impacted by LET.

### 3.2. Inhibition of ATM and DNA-Pkcs Leads to Increased DSB Persistence Following Low- and High-LET PBT

**Figure 2:**
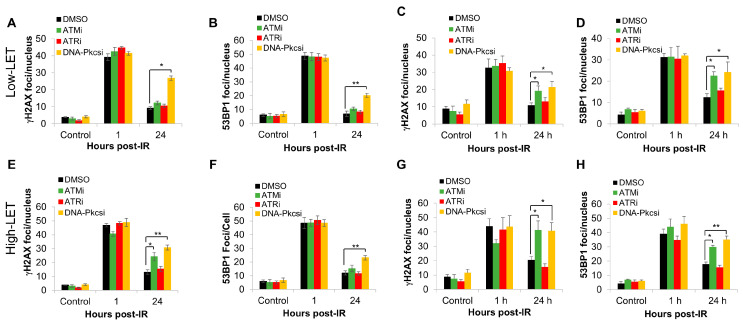
Inhibition of ATM and DNA-Pkcs causes increased persistence of γH2AX and 53BP1 foci in response to both low- and relatively high-LET PBT. (**A**,**B**,**E**,**F**) FaDu, and (**C**,**D**,**G**,**H**) A253 cells were treated with inhibitors against ATM (10 nM), ATR (1 µM) and DNA-Pkcs (1 µM) or using DMSO as a vehicle-only control for 1 h prior to irradiation. Following this, cells were treated with 4 Gy (**A**–**D**) low-LET PBT or (**E**–**H**) relatively high-LET PBT and γH2AX or 53BP1 foci measured at various time points post-irradiation by immunofluorescence microscopy. Shown is the mean foci/nucleus ± S.D. * *p* < 0.05, ** *p* < 0.01 as analysed by a one sample *t*-test.

We analysed the levels of γH2AX and 53BP1 foci, as surrogate markers of DSBs, following the protein kinase inhibitors in combination with PBT in FaDu and A253 cell lines. As expected, we observed a significant persistence in the levels of γH2AX foci at 24 h post-irradiation with low-LET PBT in the presence of DNA-Pkcs inhibitor versus the DMSO control in both cell lines, whereas with ATM inhibition, significant γH2AX foci persistence was only seen in A253 cells (Figure 2A,C and Figure S6). In contrast, ATR inhibition had no major impact on the levels of γH2AX foci at 24 h post-irradiation in either cell line. Similar data were acquired through analysis of 53BP1 foci as an indicative marker of NHEJ. Here, there was significant persistence in the numbers of foci at 24 h post-irradiation with DNA-Pkcs inhibition in both FaDu and A253 cells, whereas following ATM inhibition, 53BP1 foci levels were only persistent in A253 cells (Figure 2B,D and Figure S7). Following relatively high-LET PBT, the numbers of γH2AX foci were comparatively higher at 24 h post-irradiation compared to the same time point using low-LET PBT, indicative of DSB sites that were more difficult to repair. However, the impact of the inhibitors showed a similar trend, whereby inhibition of DNA-Pkcs caused elevated levels of γH2AX foci at 24 h post-irradiation versus the DMSO control indicative of DSB persistence in both FaDu and A253. Interestingly under these irradiation conditions, ATM inhibition also led to significant persistence of γH2AX foci in both cell lines. In contrast, inhibition of ATR had relatively little impact on γH2AX foci levels at 24 h post-irradiation compared to the DMSO control (Figure 2E,G). Similar observations were seen with 53BP1 foci, suggesting that ATM and DNA-PKcs inhibition lead to delays in DSB repair (Figure 2F,H).

**Figure 3:**
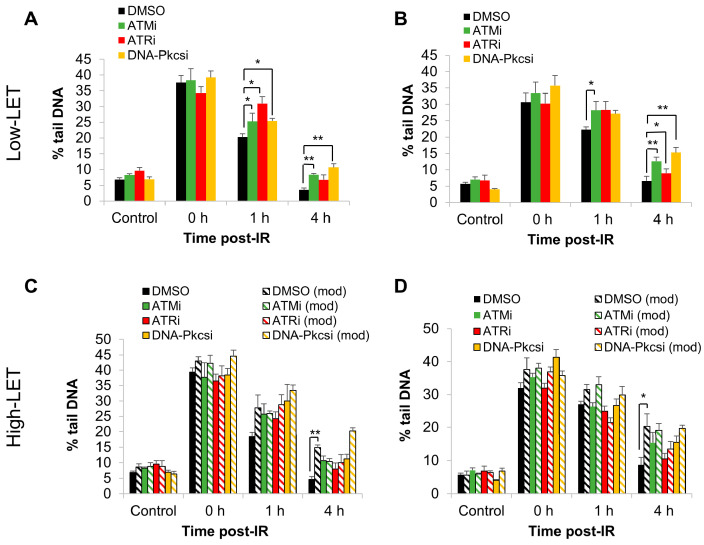
Inhibition of ATM, ATR and DNA-Pkcs causes increased persistence of DSBs following both low- and relatively high-LET PBT. (**A**,**D**) FaDu and (**B**,**D**) A253 cells were incubated with inhibitors against ATM (10 nM), ATR (1 µM) and DNA-Pkcs (1 µM) or DMSO as a vehicle-only control for 1 h prior to irradiation. Following this, cells were treated with 4 Gy (**A**,**B**) low-LET PBT or (**C**,**D**) relatively high-LET PBT. Levels of DNA DSBs (solid bars) and CDD (indicated as mod.; hatched bars) were measured at time points post-irradiation by the enzyme-modified neutral comet assay. Shown is the mean % tail DNA ± S.D. * *p* < 0.05, ** *p* < 0.01 as analysed by a one-sample *t*-test.

We then utilised the enzyme-modified neutral comet assay as a more accurate assay to directly detect and measure DNA DSBs as well as CDD (without and with enzyme modification, respectively) following the treatments. Supporting the γH2AX/53BP1 foci data, we observed that treatment with inhibitors of ATM and DNA-Pkcs significantly reduced DSB repair efficiency compared to DMSO control cells 4 h post-irradiation with low-LET PBT in both FaDu and A253 cells. ATR inhibition, in comparison, had less impact and only caused significant DSB persistence in A253 cells (Figure 3A,B, Figures S8 and S9). Similar observations were seen in FaDu and A253 cells in response to relatively high-LET PBT without enzyme modification to reveal the levels of DSBs (Figure 3C,D, compare solid bars). With enzyme modification that reveals CDD, the level of % tail DNA was notably higher in FaDu and A253 cells in the DMSO controls at 0, 1 and 4 h post-irradiation treated with high-LET PBT compared to without enzyme modification, demonstrating as expected the presence of CDD. Indeed, the persistence of DNA damage at 4 h post-irradiation was statistically different (Figure 3C,D, compare solid black and hatched bars). Despite this, the impact of the three inhibitors versus the DMSO control at either 1 or 4 h post-irradiation appeared similar with respect to the levels of CDD (Figure 3C,D, compare solid and hatched bars), indicating that kinase inhibition has no significant impact on the repair of the damage.

### 3.3. Inhibition of ATM and DNA-Pkcs Enhances Sensitivity of Patient-Derived HNSCC Organoids to Low and High-LET PBT

**Figure 4:**
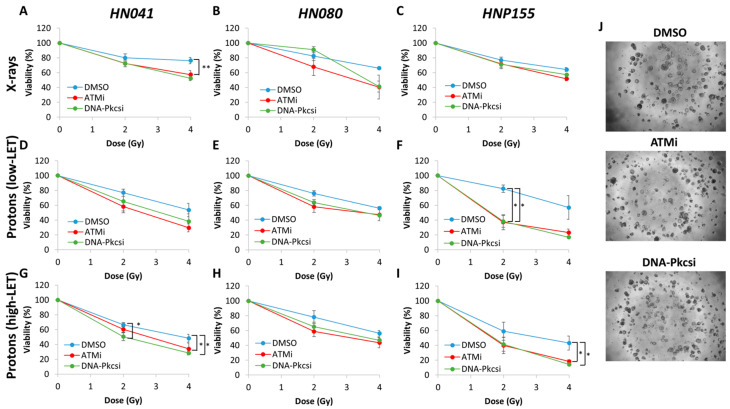
Inhibition of ATM and DNA-Pkcs leads to reduced viability of patient-derived organoids of HNSCC in response to both low- and relatively high-LET PBT. (**A**) HN041, (**B**) HN080 and (**C**) HNP155 organoids were grown for 5 days in 96-well black-walled plates and then treated with inhibitors targeting ATM (0.2 µM) or DNA-Pkcs (2 µM) or DMSO as a vehicle-only control for 4 h. Organoids were irradiated with increasing doses of either (**A**–**C**) X-rays, (**D**–**F**) low-LET PBT or (**G**–**I**) relatively high-LET PBT, and after a further 4 days, organoid viability was determined using 3D Cell-Titer Glo. Shown is the mean organoid viability ± S.E., analysed from three biologically independent experiments. * *p* < 0.05, ** *p* < 0.01 as analysed by a one-sample *t*-test. (**J**) Shown are representative images of HN041 organoids following 4 Gy low-LET PBT.

We utilised patient-derived organoids as a more appropriate model to examine the impact of inhibitors of ATM and DNA-Pkcs in combination with PBT (as the combinations that produced the most marked radiosensitisation in cell lines; Figure 1A–H and Table 2) that could replicate the patient tumour response to treatment. For completeness, we also analysed the responses of these models to X-ray irradiation in combination with the inhibitors. We discovered that the three different HNSCC organoid models displayed moderately increased sensitivity to inhibitors against ATM and DNA-Pkcs following X-ray irradiation (Figure 4A–C). HN041 only was significantly radiosensitised to the inhibitors at a 4 Gy dose. Interestingly, the inhibitors showed a marked enhancement in effectiveness in combination with PBT. Both inhibitors of ATM and DNA-PKcs were able to increase the radiosensitivity of HN041 and HNP155 to low-LET PBT, which was only significant in the latter model (Figure 4D–F,J). However, both inhibitors caused significantly enhanced radiosensitivity to high-LET PBT (Figure 4G–I). It is worth noting that the HNP155 model appeared specifically sensitive to these treatment combinations with PBT, which was different from the response to X-rays. HN080 appeared the model most resistant to treatment, where only moderate increases in radiosensitivity were observed across the different radiation modalities.

## 4. Discussion

PBT is a precision-targeted radiotherapy technique that is increasingly being used for treating patients with HNSCC, where it increases the sparing of normal tissues and organs at risk. This can therefore reduce the adverse side effect profile commonly observed with conventional X-ray radiotherapy, as recently demonstrated in a clinical trial with oropharyngeal patients [24]. However, there are still biological and clinical uncertainties with PBT due to the increases in ionisation densities/LET at and around the Bragg peak. Additionally, tumour radioresistance and recurrence is a common problem requiring the need for strategies leading to optimal radiosensitisation. The therapeutic effect of PBT, similar to X-rays, is largely driven through the formation and persistence of DNA damage, particularly DSBs and CDD. Indeed, the increasing LET generated via the Bragg peak will lead to enhancements in the levels and complexity of CDD that drive biological effectiveness. Given this, it is clear that proteins acting within the cellular DDR remain as targets for drugs/inhibitors that can be utilised to enhance tumour cell killing by PBT. ATM, ATR and DNA-Pkcs are the major kinases that coordinate the signalling and repair of DNA damage, and evidence suggests that inhibiting these kinases can radiosensitise HNSCC to X-rays [9,13,14]. Predictably, this strategy would also be effective in combination with PBT as an alternative source of radiotherapy; however, this evidence and the contribution of proton LET to tumour radiosensitisation is unclear. Here in this study, we compared the effect of potent and specific inhibitors of ATM, ATR and DNA-Pkcs on the radiosensitisation of both monolayer and patient-derived organoid models of HNSCC following PBT at both low and relatively high LET.

We discovered that ATM or DNA-Pkcs inhibitors can synergise with PBT at both low and relatively high LET in decreasing the clonogenic survival of several HNSCC cells. Inhibitors against ATM and DNA-Pkcs were the most effective in reducing clonogenic survival of the four HNSCC cell lines used in this study in combination with low- and relatively high-LET PBT. Inhibition of ATR was less effective in radiosensitising cells to low-LET PBT and showed no impact following relatively high-LET PBT. Based on this, we focussed on the radiosensitising potential of the ATM and DNA-Pkcs inhibitors in patient-derived organoids as a more accurate model of the response of patient tumours to radiotherapy. Here, we demonstrated that both inhibitors were able to significantly reduce the viability of three HNSCC organoid models following low- and relatively high-LET PBT, as well as X-rays, although the specific responses were variable. Interestingly, in one HNSCC organoid model (HNP155), this was markedly sensitive to the combination of ATM or DNA-Pkcs inhibitors with PBT compared to X-rays, whereas another model (HN080) was relatively insensitive and where further investigations are needed to identify the underlying factors responsible for this. Nevertheless, these findings are in support of our previous study focussed specifically on low-LET X-rays and PBT [19] and collectively support the fact that inhibition of ATM or DNA-Pkcs is able to potentiate the cell killing effects of PBT, including with increasing LET.

We appreciate that our study in analysing the responses of HNSCC cells in the presence of the inhibitors is limited to just two different positions, and therefore LETs, relative to the Bragg peak. A range of broader LET responses therefore needs to be exploited. Supporting evidence of our findings from the literature is, however, scarce particularly in relation to increasing proton LET. However, the DNA-Pkc inhibitors IC87361 and KU-57788 have previously been demonstrated to cause enhanced sensitivity of HNSCC cell lines following low-LET X-rays and PBT [16,25], and the ATM inhibitor GSK635416A similarly has been proven to enhance radiosensitivity of several HNSCC cell lines following X-rays [26]. It is interesting to note that a number of studies have demonstrated that different ATR inhibitors can radiosensitise HNSCC cells in response to X-rays [15,17,18,25,27], although our evidence actually supports ATM or DNA-Pkcs as the strongest targets for radiosensitisation. The reason for the lack of radiosensitisation using ATR inhibition in our study is unclear but could relate to the different cell models used and their capacity or reliance on HR driven by ATR for DSB repair. We also noted observable toxicity of the ATR inhibitor treatment alone on HNSCC cells; therefore, the level of inhibitor used had to be carefully balanced.

We correlated the effects of kinase inhibition of ATM and DNA-Pkcs on radiosensitisation of HNSCC cells with persistence in DNA DSBs and CDD using enzyme-modified neutral comet assays, which was supported through using γH2AX and 53BP1 foci as surrogate markers of DSBs. This is not unsurprising and supports other reported evidence of the specificity of AZD1390 and AZD7648 in the inhibition of the respective kinase leading to suppression of DSB repair. We also previously demonstrated that the combinations of these inhibitors with low-LET X-rays and PBT leads to significantly increased levels of micronuclei [19], indicating that mitotic catastrophe is the likely mechanism through which cell death is achieved. Taken together, our findings provide further support that inhibition of ATM and DNA-Pkcs, as the major protein kinases involved in the signalling and repair of DNA DSBs, can enhance the radiosensitivity of HNSCC cells following PBT independent of LET. As ATM and DNA-Pkcs are largely employed in NHEJ, this supports the evidence in the literature that this is the primary DSB repair pathway employed following PBT (including now of increasing LET), as well as X-rays [28,29]. This would, however, contradict evidence suggesting that PBT shows a greater reliance on the HR pathway coordinated by ATR for repairing DSBs [30,31]. It is worth noting that these previous studies utilised HR-deficient Chinese Hamster Ovary cells and A549 cells depleted of RAD51, compared to the HNSCC and ATR inhibitor (AZD6738) used in our study, suggesting either cell or experiment-specific differences that may explain these effects. Despite this, we aim to further explore the potential for ATM and DNA-Pkcs inhibitors to radiosensitise HNSCC tumours in vivo. Here, we will utilise patient-derived xenografts using immunocompromised mice to demonstrate that the combination of either ATM or DNA-Pkcs inhibitors with PBT can suppress the growth of HNSCC tumours in vivo. We will also use this model to confirm the accumulation of DNA DSBs in the tumour tissue through γH2AX/53BP1 analysis using immunohistochemistry analysis but also to assess any toxicity of the treatments on the animals. Collectively, these experiments will provide further supporting evidence that this strategy has the greatest potential to optimise PBT treatments for HNSCC patients in the clinic.

Of particular note for further ongoing investigations is the need to understand the impact of hypoxia (0.1–1% oxygen) on the tumour responses to PBT in combination with the DNA repair inhibitors, which could be different from those observed in normoxia in the current study. This is specifically important for solid tumours of the head and neck where hypoxia forms a significant barrier to effective treatment, particularly with low-LET radiotherapy. Additionally, it is necessary to fully appreciate the impact of the DNA repair inhibitors on the normal cells and tissues within the head and neck, despite the proposed utilisation of precision-targeted PBT. This is needed to ensure that there is no overt normal tissue toxicity associated with the treatment prior to clinical translation. It should be noted that our study utilised single radiation doses in combination with the inhibitors to examine the effects on HNSCC models, whereas dose fractionation is used clinically. This is aspect is challenging in cell models that generally do not tolerate multiple treatment doses well, and which needs to be explored in other more treatment-resistant models such as organoids and tumours grown in vivo. It would also be interesting to examine whether current chemotherapeutics, such as cisplatin, or other therapies such as immunotherapies are able to synergise with the radiation–DNA repair combinations to produce the most optimal tumour cell killing ability whilst sparing the normal tissue. Altogether, these areas of investigation would require additional preclinical evidence, including the use of HNSCC cells and organoids in this study, before moving forward with in vivo experiments using animals. We also accept that our data would need reproducing using a clinical proton system (e.g., 250 MeV) that operates within a higher energy range, compared to the relatively lower energies used in our study for examining radiobiology.

## Figures and Tables

**Table 1:** Statistical analysis of clonogenic survival of HNSCC cell lines following ATM, ATR or DNA-Pkcs inhibition with low- or relatively high-LET PBT.

Treatment	FaDu	UMSCC12	A253	UMSCC17A
Low-LET ATMi	*p* < 0.0008	*p* < 1.1 × 10^−6^	*p* = 0.21	*p* < 0.002
Low-LET ATRi	*p* = 0.16	*p* < 0.04	*p* < 0.05	*p* = 0.08
Low-LET DNA-PKcsi	*p* < 2.58 × 10^−8^	*p* < 2.7 × 10^−11^	*p* < 0.002	*p* < 0.0006
High-LET ATMi	*p* = 0.15	*p* < 2.1 × 10^−5^	*p* < 0.005	*p* < 0.03
High-LET ATRi	*p* = 0.84	*p* = 0.89	*p* = 0.60	*p* = 0.71
High-LET DNA-PKcsi	*p* < 1.1 × 10^−6^	*p* < 1.4 × 10^−11^	*p* < 0.02	*p* = 0.07

**Table 2:** Dose enhancement ratios (DER) at 37% survival for HNSCC cell lines following ATM, ATR or DNA-Pkcs inhibition with low- or relatively high-LET PBT.

Treatment	FaDu	UMSCC12	A253	UMSCC17A
Low-LET ATMi	1.58	1.15	1.53	1.41
Low-LET ATRi	1.15	0.79	1.77	1.25
Low-LET DNA-PKcsi	2.69	2.66	1.76	2.31
High-LET ATMi	1.41	1.43	1.53	1.66
High-LET ATRi	0.90	0.92	1.12	1.11
High-LET DNA-PKcsi	1.93	2.25	1.50	1.50

## Data Availability

Source data are provided within this paper. Any other data will be made available from the corresponding author upon reasonable request.

## Supplementary Materials

The following supporting information can be downloaded at: https://www.mdpi.com/article/10.3390/cells15100879/s1.
