# Supplementary material for: Radiosensitisation of Head and Neck Cancer Cells to Protons of Increasing LET Through Targeting DNA Double Strand Break Repair
Source: Cells. 2026 May 12;15(10):879. doi: 10.3390/cells15100879 (PMC13205111; doi:10.3390/cells15100879)
Supplement: Supplementary file 1 [file cells-15-00879-s001.zip › cells-4255558-supplementary.pdf]

SUPPLEMENTARY DATA

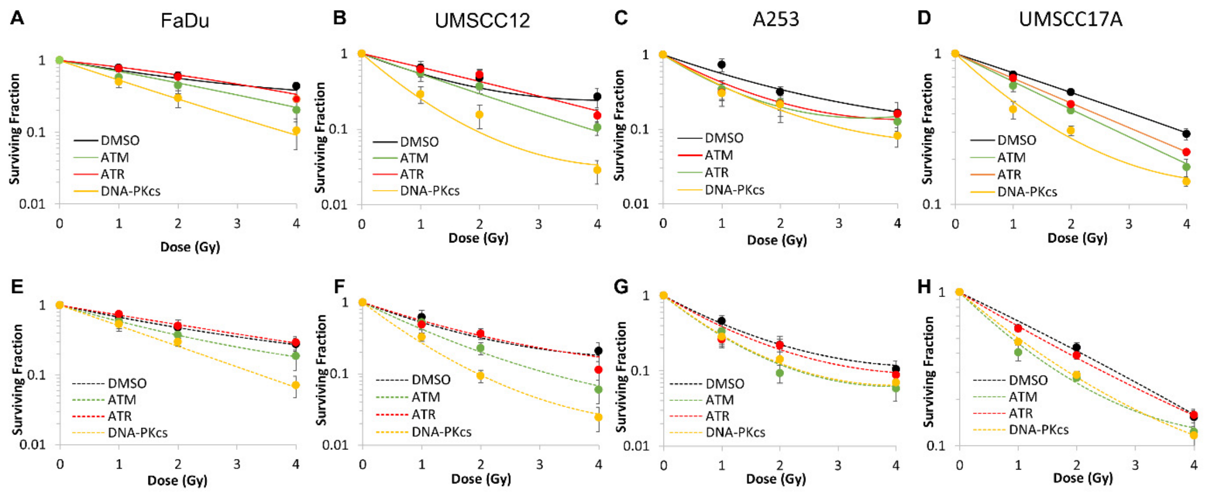

Supplementary Figure S1. Inhibition of ATM, ATR and DNA-Pkcs leads to increased radiosensitivity of HNSCC cells to both low and relatively high-LET PBT. (A) FaDu, (B) UMSCC12, (C) A253 or (D) UMSCC17A cells were treated with either 10 nM AZD1390, 1 μM AZD6738, 1 μM AZD7648 or DMSO for 1 h prior to exposure to (A-D) low-LET PBT or (E-H) relatively high-LET PBT, and clonogenic survival of the cells was analysed from three biologically independent experiments. Shown is the data fitted according to the linear quadratic (LQ) model.

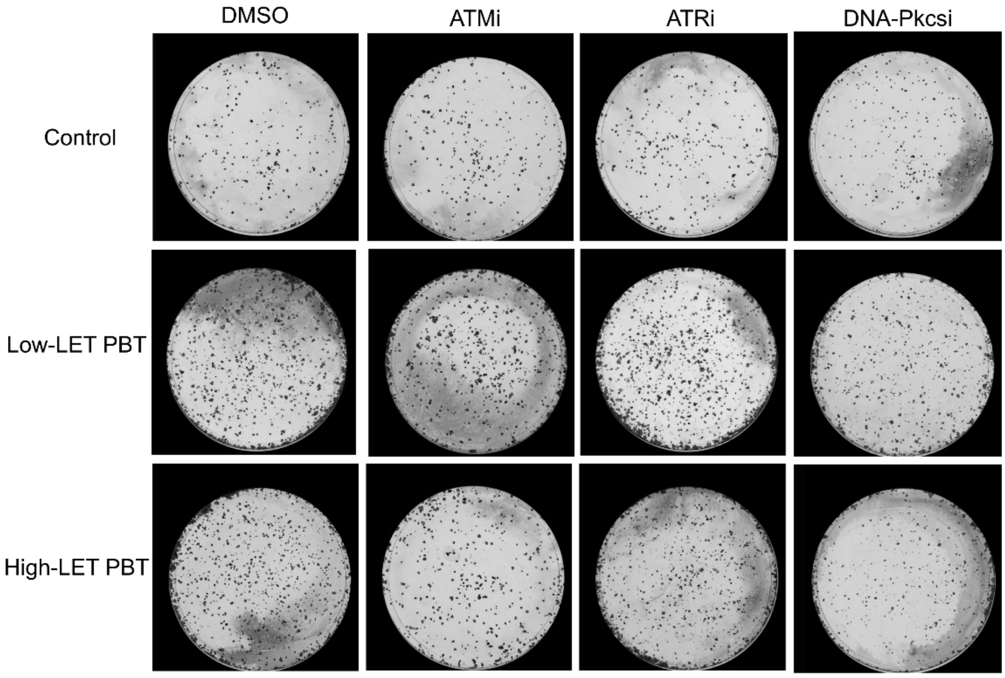

Supplementary Figure S2. Inhibition of ATM, ATR and DNA-Pkcs leads to increased radiosensitivity of FaDu cells to both low and relatively high-LET PBT. FaDu cells were treated with either 10 nM AZD1390, 1 μM AZD6738, 1 μM AZD7648 or DMSO for 1 h prior to exposure to low-LET PBT or relatively high-LET PBT. Shown are representative images of colonies formed in the absence (Control) and presence of 4 Gy radiation dose (where the numbers of cells seeded was four times higher).

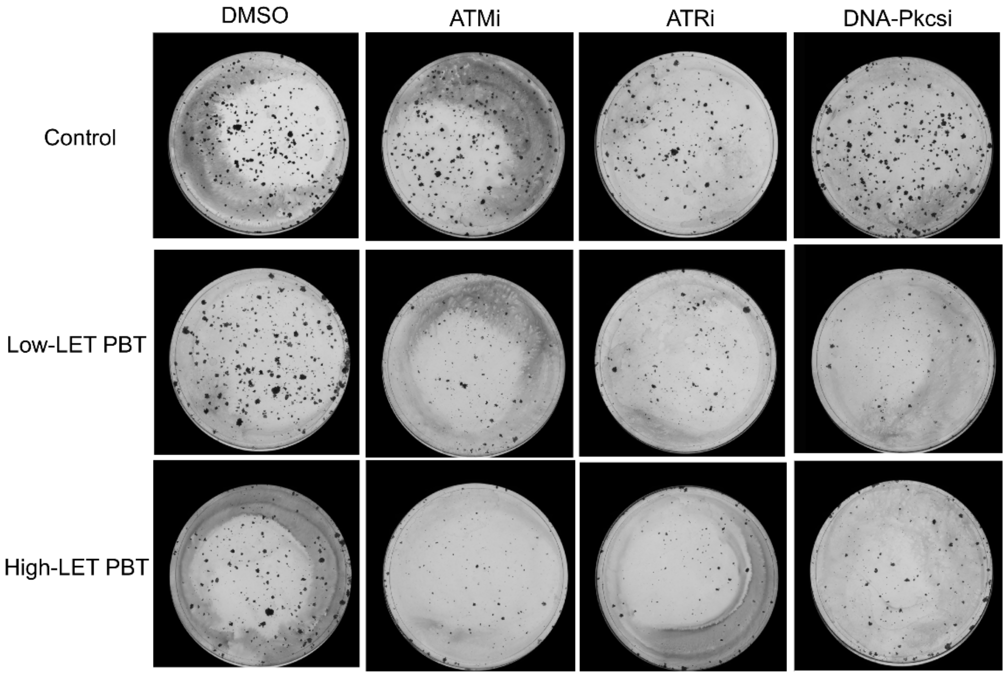

Supplementary Figure S3. Inhibition of ATM, ATR and DNA-Pkcs leads to increased radiosensitivity of A253 cells to both low and relatively high-LET PBT. A253 cells were treated with either 10 nM AZD1390, 1 μM AZD6738, 1 μM AZD7648 or DMSO for 1 h prior to exposure to low-LET PBT or relatively high-LET PBT. Shown are representative images of colonies formed in the absence (Control) and presence of 4 Gy radiation dose (where the numbers of cells seeded was four times higher).

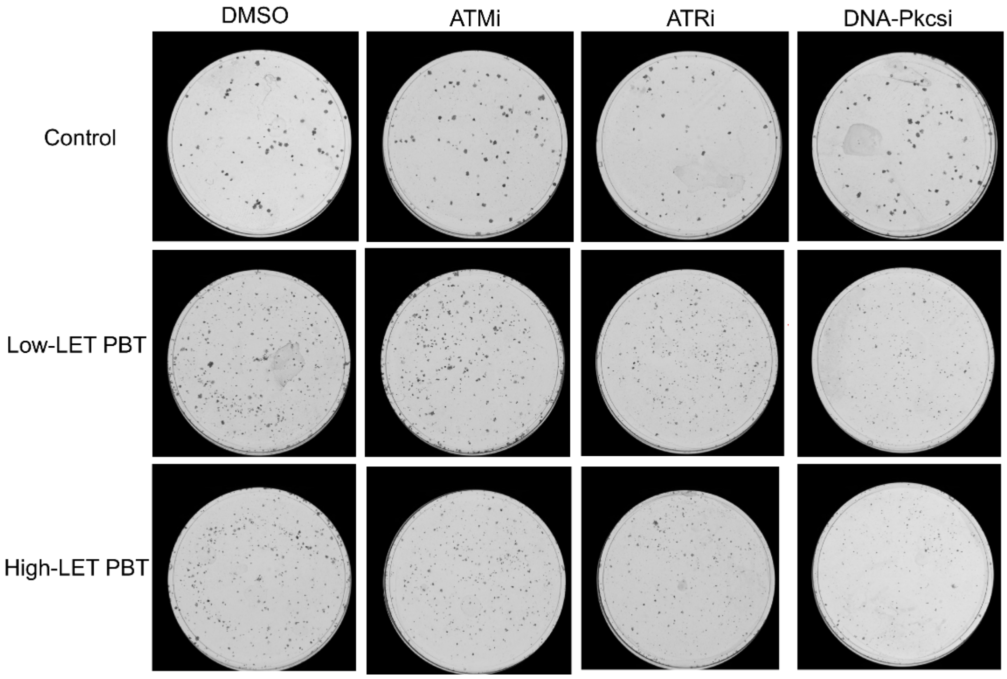

Supplementary Figure S4. Inhibition of ATM, ATR and DNA-Pkcs leads to increased radiosensitivity of UMSCC12 cells to both low and relatively high-LET PBT. UMSCC12 cells were treated with either 10 nM AZD1390, 1 μM AZD6738, 1 μM AZD7648 or DMSO for 1 h prior to exposure to low-LET PBT or relatively high-LET PBT. Shown are representative images of colonies formed in the absence (Control) and presence of 4 Gy radiation dose (where the numbers of cells seeded was four times higher).

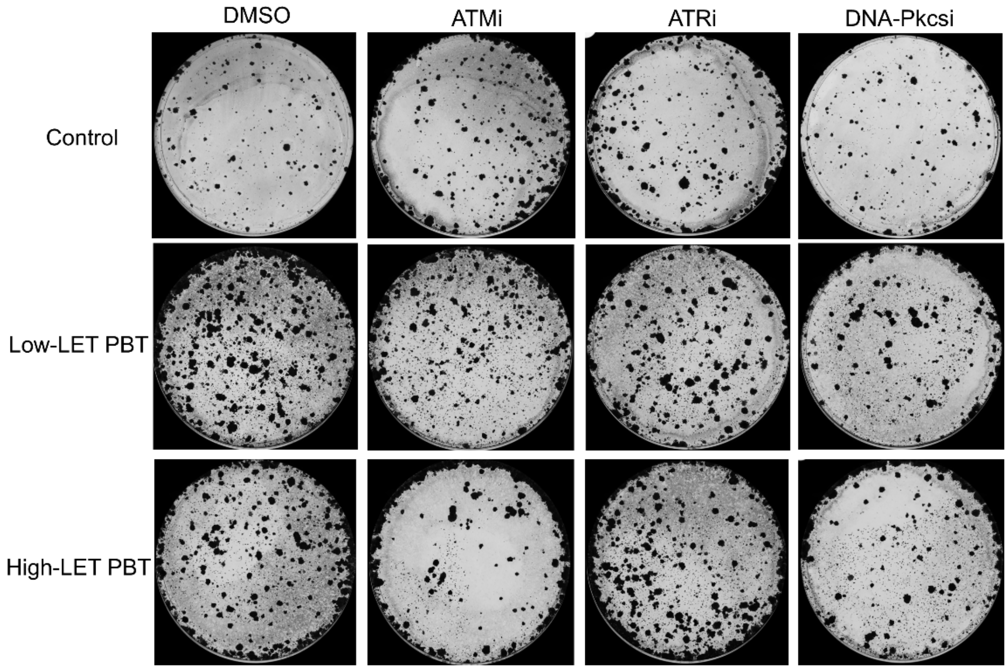

Supplementary Figure S5. Inhibition of ATM, ATR and DNA-Pkcs leads to increased radiosensitivity of UMSCC17A cells to both low and relatively high-LET PBT. UMSCC17A cells were treated with either 10 nM AZD1390, 1 μM AZD6738, 1 μM AZD7648 or DMSO for 1 h prior to exposure to low-LET PBT or relatively high-LET PBT. Shown are representative images of colonies formed in the absence (Control) and presence of 4 Gy radiation dose (where the numbers of cells seeded was four times higher).

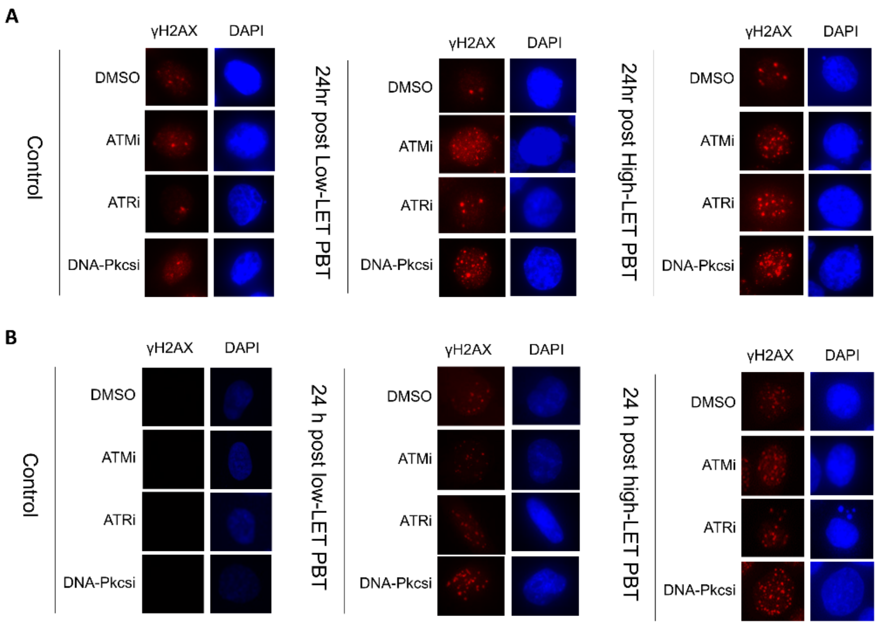

Supplementary Figure S6. Inhibition of ATM and DNA-Pkcs leads to increased persistence of radiation-induced γH2AX foci. (A) FaDu or (B) A253 cells were treated with either 10 nM AZD1390, 1 µM AZD6738, 1 µM AZD7648 or DMSO for 1 h prior to exposure to low-LET PBT or relatively high-LET PBT. DNA DSB damage was measured at various timepoints post-irradiation using γH2AX foci through immunofluorescence microscopy. Shown are representative images for the unirradiated controls, plus 24 h post-irradiation.

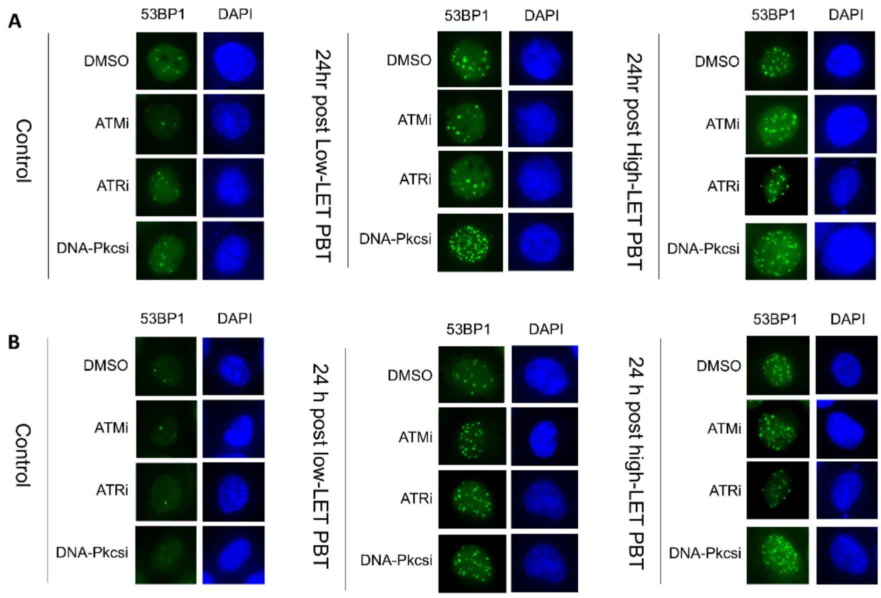

Supplementary Figure S7. Inhibition of ATM and DNA-Pkcs leads to increased persistence of radiation-induced 53BP1 foci. (A) FaDu or (B) A253 cells were treated with either 10 nM AZD1390, 1 µM AZD6738, 1 µM AZD7648 or DMSO for 1 h prior to exposure to low-LET PBT or relatively high-LET PBT. DNA DSB damage was measured at various timepoints post-irradiation using 53BP1 foci through immunofluorescence microscopy. Shown are representative images for the unirradiated controls, plus 24 h post-irradiation.

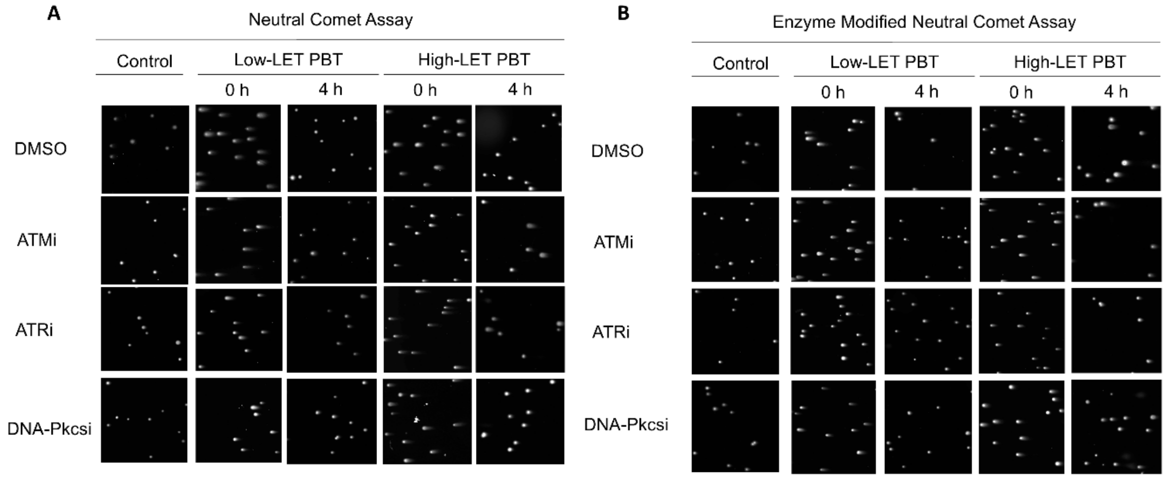

Supplementary Figure S8. Inhibition of ATM and DNA-Pkcs leads to increased persistence of radiation-induced DSBs in FaDu cells. FaDu cells were treated with either 10 nM AZD1390, 1 µM AZD6738, 1 µM AZD7648 or DMSO for 1 h prior to exposure to low-LET PBT or relatively high-LET PBT. DNA DSB and CDD damage was measured at various timepoints post-irradiation using the enzyme-modified neutral comet assay in (A) the absence, or (B) the presence of enzyme modification. Shown are representative images for the unirradiated controls, plus 0 and 4 h post-irradiation.

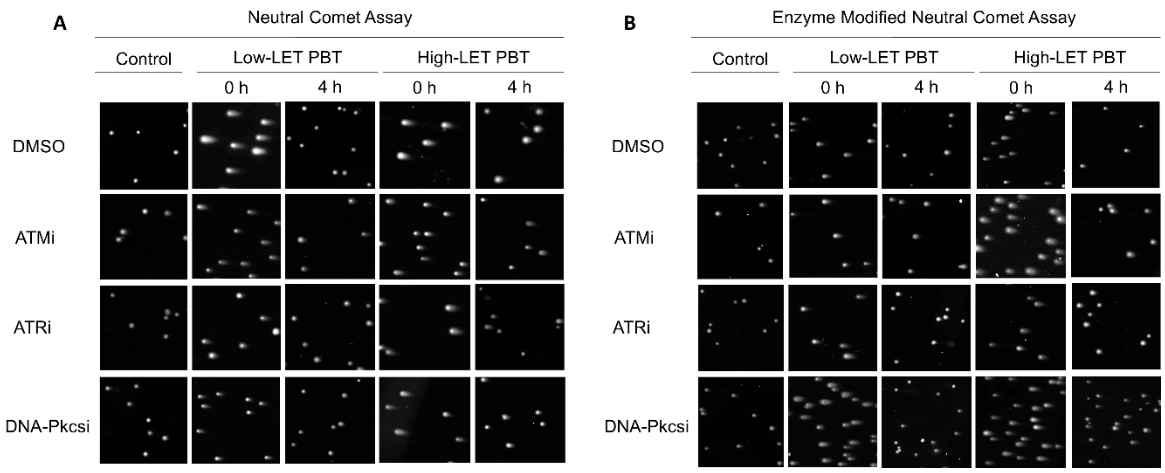

Supplementary Figure S9. Inhibition of ATM and DNA-Pkcs leads to increased persistence of radiation-induced DSBs in A253 cells. A253 cells were treated with either 10 nM AZD1390, 1 µM AZD6738, 1 µM AZD7648 or DMSO for 1 h prior to exposure to low-LET PBT or relatively high-LET PBT. DNA DSB and CDD damage was measured at various timepoints post-irradiation using the enzyme-modified neutral comet assay in (A) the absence, or (B) the presence of enzyme modification. Shown are representative images for the unirradiated controls, plus 0 and 4 h post-irradiation.

Supplementary Table S1. Characteristics of HNSCC organoids

	HN041	HN080	HNP155
Patient age	62	65	57
Subsite	hypopharynx	oral cavity	tongue
Tumour stage	IVA	IVA	IVB
HPV status	negative	negative	negative
TP53 status	mutant	mutant	mutant
EGFR status	wild type	wild type	amplification
CDKN2A status	wild type	mutant	wild type
PIKC3A status	amplification	wild type	wild type
